# Active travel to schools in Wales

**DOI:** 10.23889/ijpds.v8i2.2313

**Published:** 2023-09-14

**Authors:** Alex Sutherland

**Affiliations:** 1 Research Design UK, Cambridge, United Kingdom

## Objectives

This project combined administrative data with completed randomised controlled trials to understand whether educational interventions led to additional benefits or costs beyond those initially evaluated. The purpose of the project was to demonstrate that this can be done, the value of such work, and what needs to happen next to embed such approaches.

## Methods

We reviewed the Education Endowment Foundation trials database for studies that could plausibly link to other outcomes: exclusion / suspension from school, criminal conviction, or being Not in Education, Employment or Training (NEET). After shortlisting we intended to link at least one trial to different administrative datasets containing these outcomes.

We undertook three other tasks:

1. Understanding barriers to data sharing within government from a behavioural perspective and proposed solutions to those challenges.
2. Reviewing theories of change for some interventions to assess whether/how there could be pathways to the outcomes we were looking at.
3. Asking intervention developers and evaluators for their predictions and rationales for possible impacts on other outcomes, without them knowing the results from reanalysis.

## Results

We were successful in linking more than 30 completed trials to new outcome data. In some instances we were able to recover more outcome data than the original study through the use of administrative data. We then reanalysed these trials to assess the impact of these interventions on alternative outcomes.

We also developed an approach to creating low-fidelity synthetic data to allow for more and easier data sharing by data owners, as well as proposing an asymmetric approach to linkage that can speed up results.

## Conclusion

In some instances, seemingly negative and positive impacts of interventions that would otherwise not be known were observed. Beyond the results of the reanalysis, which prompted recommendations to a major funder, the project contributed to shaping processes for accessing administrative data, started conversations within government and other data owners about synthetic data, as well as providing a demonstration project for data linkage and reuse.

